# January 2015 Issue, vol. 104 (1), pages 32-44

**DOI:** 10.5935/abc.20150138

**Published:** 2015-10

The original article "Cost-Effectiveness of High, Moderate and Low-Dose Statins in the Prevention of Vascular Events in the Brazilian Public Health System" published in the January 2015 issue of the Brazilian Archives of Cardiology [Arq Bras Cardiol. 2015; 104 (1): 32-44], was published in the Portuguese version as "Effectiveness of High, Moderate and Low-Dose Statins in the Prevention of Vascular Events in the Brazilian Public Health System". A correction was made in the Portuguese version.

